# Mortality of the U. S.

**Published:** 1872-06

**Authors:** 


Mortality of the United States by states and territories, with distinctions of sex and percentage of deaths to population from the censuses of 1870, 1860, and 1850. Washington 1872 government printing office.

The value of these tables will be readily understood, when we are told that they distribute nearly one half a million of deaths, by age, sex, color, nativity, occupation, and month of death, according to disease. These tables are to be preceeded in the full volume by the percentage tables and remarks; they will also be followed by the statistics of the Insane, Blind, Dumb, &c. &c. We have been interested and instructed in looking over these tables and have no doubt, but that they will be of much value to the medical profession, when issued in their complete form.

